# Hatching delays in great tits and blue tits in response to an extreme cold spell: a long-term study

**DOI:** 10.1007/s00484-018-1541-3

**Published:** 2018-04-17

**Authors:** Michał Glądalski, Mirosława Bańbura, Adam Kaliński, Marcin Markowski, Joanna Skwarska, Jarosław Wawrzyniak, Piotr Zieliński, Jerzy Bańbura

**Affiliations:** 1 Department of Experimental Zoology and Evolutionary Biology, Faculty of Biology and Environmental Protection, University of Łódź, Banacha 12/16, 90-237 Łódź, Poland; 2 Museum of Natural History, Faculty of Biology and Environmental Protection, University of Łódź, Kilińskiego 101, 90-011 Łódź, Poland; 3 Department of Ecology and Vertebrate Zoology, Faculty of Biology and Environmental Protection, University of Łódź, Banacha 12/16, 90-237 Łódź, Poland

**Keywords:** Climate change, Phenology, Laying date, Extreme weather event, Delayed breeding time

## Abstract

Variation in ambient temperature affects various life stages of organisms. It has been suggested that climate change not only implies higher global temperatures but also more unpredictable weather and more frequent extreme weather events. Temperature has a major influence on the optimal laying-incubation-hatching dates of insectivorous passerines, because it poses energetic constraints and affects the timing of food abundance. We have been studying breeding characteristics of great tits *Parus major* and blue tits *Cyanistes caeruleus* in two areas, an urban parkland and a deciduous forest, around the city of Łódź since 2002. During the egg-laying period in 2017, both tit species at both study areas faced an unusual cold spell as reflected by a sudden decrease in the mean ambient temperature to ca. 2–3 °C for about 5 days, which caused mean hatching delays of up to 6 days. Since flexibility of behavior plays a major role in adjusting to unpredictable weather conditions, examining its limits may be an important goal for future research.

## Introduction

Variation in ambient temperature affects various life stages of organisms (Stearns 1992; Mainwaring and Hartley 2016; Rodríguez et al. 2016; Bleu et al. 2017; Vaugoyeau et al. 2017). It has a large influence on the optimal laying-incubation-hatching dates of insectivorous passerines, because it affects the timing of food abundance (Perrins 1991; Hinks et al. 2015). A combination of warm weather and mild rainfall in spring provides good conditions which enable the development of plants and rich arthropod communities, while low temperature slows down these processes. Clutch initiation date in tits is characterized by wide phenotypic plasticity and it largely depends on the temperatures directly before the laying of the first egg. The moment of initiating a clutch by a single tit female may differ more than 3 weeks between breeding seasons depending on the temperatures (Glądalski et al. 2014, 2016a; Wesołowski et al. 2016) but also on the habitat type (Blondel et al. 1993; Massa et al. 2011). When the temperatures are appropriate, females start producing eggs (in tits one per day) and then, if there is a sudden temperature drop, they may delay the moment of laying the next eggs or delay the moment of starting incubation. It is rather difficult to pause the incubation for more than a few hours without losing the clutch (Lee and Lima 2017). Therefore, delays in hatching usually occur when females are faced with a sudden cold spell (García-Navas and Sanz 2011; Kluen et al. 2011; Tomás 2015) and may be considered as beneficial, when they allow for better synchronization between food demands of nestlings and the peak of caterpillar availability (Monrós et al. 1998; Cresswell and McCleery 2003). Females may also accelerate hatching, by starting incubation before producing their last eggs, when conditions are improving. Additionally, laying gaps and hatching delay may be interpreted as a consequence of food shortage or increased costs of thermoregulation during egg laying period (Nilsson and Svensson 1993a, b; Cucco et al. 2017). Little is known about the flexibility of the hatching delay (Naef-Daenzer et al. 2004; Kluen et al. 2011) and since phenotypic plasticity and flexibility of behavior play a major role in adjusting to unpredictable weather conditions in spring, examining their limits may be an important goal for research (Tomás 2015).

There is a need to study variation in weather characteristics before and during the breeding period in birds in order to understand the ecological implications of climate change and more frequent extreme weather events (Charmantier et al. 2008; Goodenough et al. 2011; Pipoly et al. 2013; Donnelly and Yu 2017; Marrot et al. 2017). Extreme weather events are seen as weather conditions that cause the biological response to be in the 5% of most extreme values of the response variable (Altwegg et al. 2017; van de Pol et al. 2017). As a result, the number of studies on various phenology traits has recently increased (Gaughan et al. 2017; Sheridan and Allen 2017). It was also suggested that climate change not only implies higher temperatures and global changes in precipitation, but also more frequent extreme weather events, like cold spells in spring, or warm spells during winter (Otto 2015; Buckley and Huey 2016; Bailey and van de Pol 2016; Ummenhofer and Meehl 2017). Some authors even suggest that extreme weather events may have stronger effects on wildlife populations and habitats than changes in averages (Bateman et al. 2015; Martinuzzi et al. 2016). In addition to variation in local weather conditions, the occurrence of extreme weather events also affects breeding birds (Jenouvrier 2013; Mainwaring et al. 2017). A cold snap during the breeding season may have large consequences for breeding birds (Glądalski et al. 2014, 2016a; Indykiewicz 2015; Tobolka et al. 2015). On the other hand, it was suggested that the recent extreme weather events can be treated as natural experiments that may elucidate the mechanisms by which birds adjust their phenology to fluctuating environments (Both and Visser 2005; Jentsch et al. 2007; Glądalski et al. 2016a; Altwegg et al. 2017). Fletcher et al. (2013) and Whitehouse et al. (2013) conclude that there is a need to collect long-term phenology monitoring data in order to fully understand the impacts of climate change on different species. Bauer et al. (2010) note in addition that most papers analyzing these trends do not use data from central Europe, and there is a need to fill this gap.

In 2017, a large temperature drop during breeding was noticed in many parts of Europe and caused hatching delays in many tit populations in Belgium, England, France, Germany, Hungary, the Netherlands, Sweden, and others (Massemin S. personal information, Matthysen E. personal information, Nilsson J.-Å. personal information, Santema P. personal information, Seress G. personal information, Szulkin M. personal information, Visser M. personal information—information gathered during 8th International Hole-Nesting Birds Conference, Trondheim, Norway, October 30–November 2, 2017). In 2017, great temperature drop occurred shortly after the moment of the initiation of tit breeding at our study areas. Such temperature drops may indeed be seen as natural experiments. The aim of this paper is to show effects of extreme temperature drops during breeding on hatching delays in the great tit *Parus major* and the blue tit *Cyanistes caeruleus* at an urban parkland and a deciduous forest in central Poland. We suggest that during colder weather, ecological interactions, including predator-prey interactions, change (smaller amounts of prey available), and it may lead to changes in breeding strategies—eggs of small songbirds are built from the current income of resources. Additionally, female parents may require more food for body maintenance than for eggs, and this may lead to days with no produced eggs. Therefore, we predict that hatching delay should depend on ambient temperatures during breeding and very low temperatures should increase hatching delay.

## Materials and methods

This study was carried out in 2002–2017 as part of long-term research project concerning the breeding biology of secondary hole-nesting birds occupying nestboxes near Łódź, central Poland (51° 47′ N, 19° 28′ E) (Glądalski et al. 2015, 2017; Wawrzyniak et al. 2015). Both study sites are located in two, 10-km-distant, structurally and floristically contrasting habitats, an urban parkland (51° 45′ N, 19° 24′ E) and a deciduous forest (51° 50′ N, 19° 29′ E). The urban parkland area (80 ha) consists of the zoological garden (16 ha) and the botanical garden (64 ha). This area is one of the biggest recreation and entertainment areas in Łódź (Glądalski et al. 2016b). The vegetation of the parkland area consists of a diverse mix of tree species including exotic tree species (Marciniak et al. 2007). The forest site is about 130 ha area in the center of mature mixed deciduous forest (Łagiewniki forest, 1250 ha in total), bordering on the NE suburbia of Łódź. Large parts of the forest come directly from the ancient woodland typical for this region of central Europe. Oaks (*Quercus robur* and *Q. petraea*) are predominating tree species in the forest.

Both study areas were supplied with standard wooden nestboxes (Lambrechts et al. 2010). About 200 nestboxes were set in the parkland and about 300 nestboxes were set in the forest. All the nestboxes were placed on trees (usually on oaks) at a height of about 3 m. In both study areas, distances between neighboring nestboxes were about 50 m. At the start of the breeding season, the nestbox study areas were visited every day to record nestbox occupancy, laying date, clutch size, and hatching day. In normal conditions, the female lays one egg per day in tits (Perrins 1996). In situations when we found older hatchlings, we estimated hatching day using our photographic key for age determination of nestling tits. In the case of great tits, only first clutches were analyzed—clutches that started no more than 30 days from the first clutch in a studied population during the breeding season (van Noordwijk et al. 1995). A total of 1517 (890 in the parkland and 627 in the forest) first clutches of the great tit and a total of 835 (348 in the parkland and 487 in the forest) first clutches of the blue tit were studied.

**Fig. 1:**
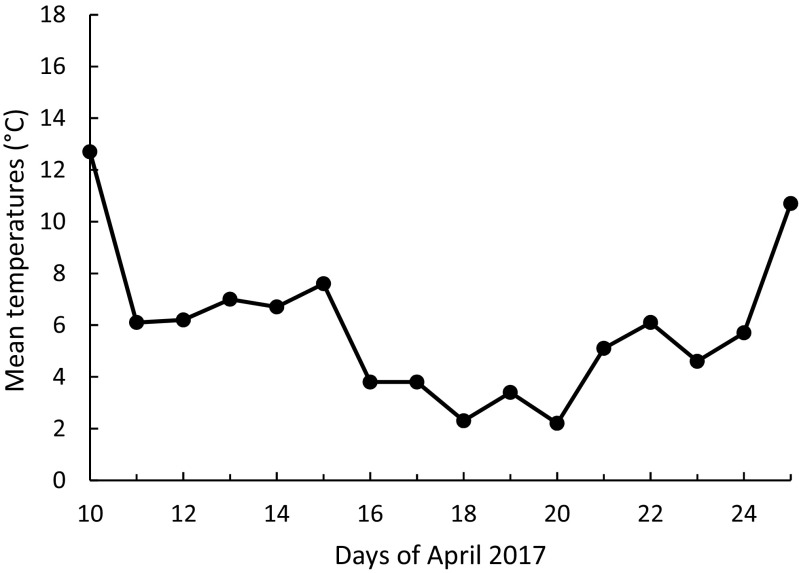
Mean temperatures for the mid-laying-early-incubating time in April 2017

**Fig. 2:**
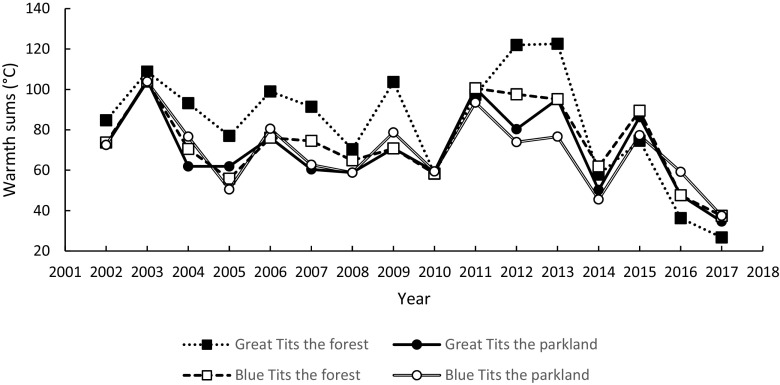
Mid-laying-early-incubating warmth sums, as the sum of the mean daily temperatures for the 7 days starting in the 4th day since the first egg date, in great tits and blue tits and in the forest study area and in the urban parkland study area (2002–2017)

**Fig. 3:**
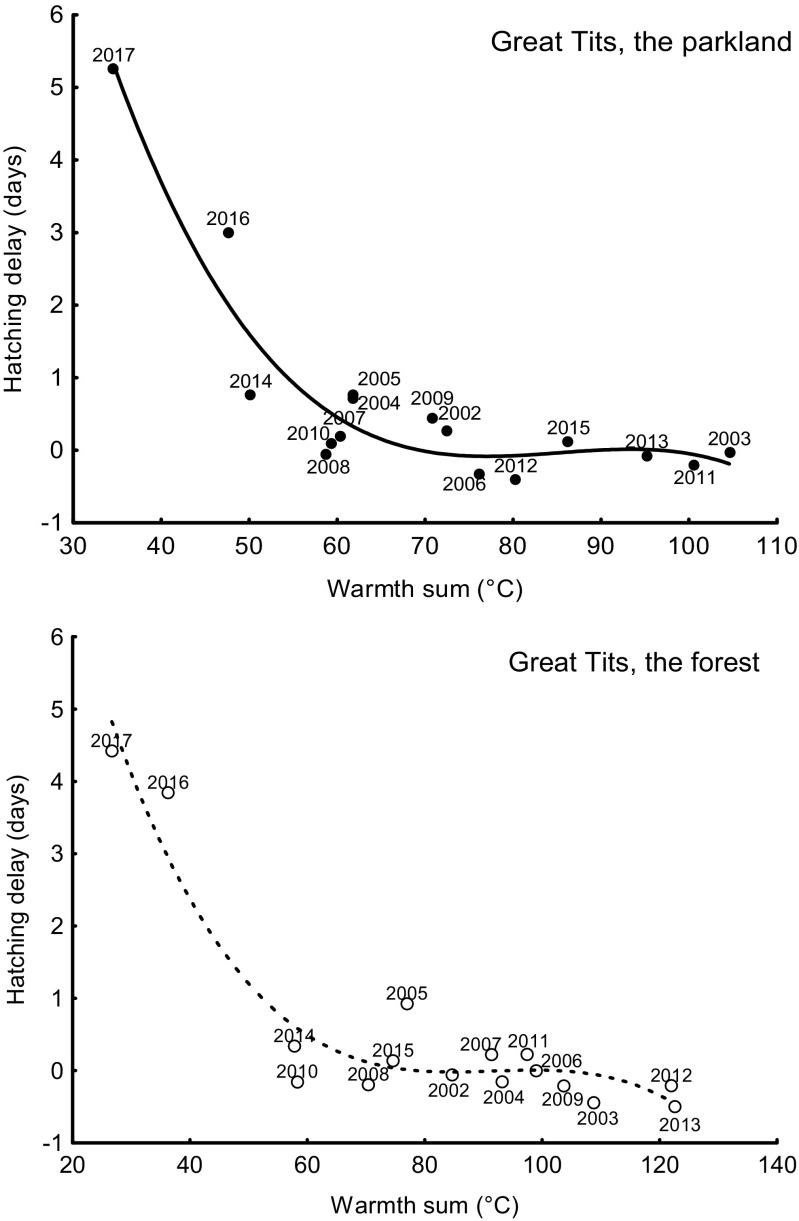
Relationship between hatching delay (days) and the sum of the mean daily temperatures for the 7 days starting on the 4th day since the first egg date (warmth sum, °C) for great tits in the urban parkland area (cubic, filled circles) and in the forest area (cubic, open circles) (seasons 2002–2017)

**Fig. 4:**
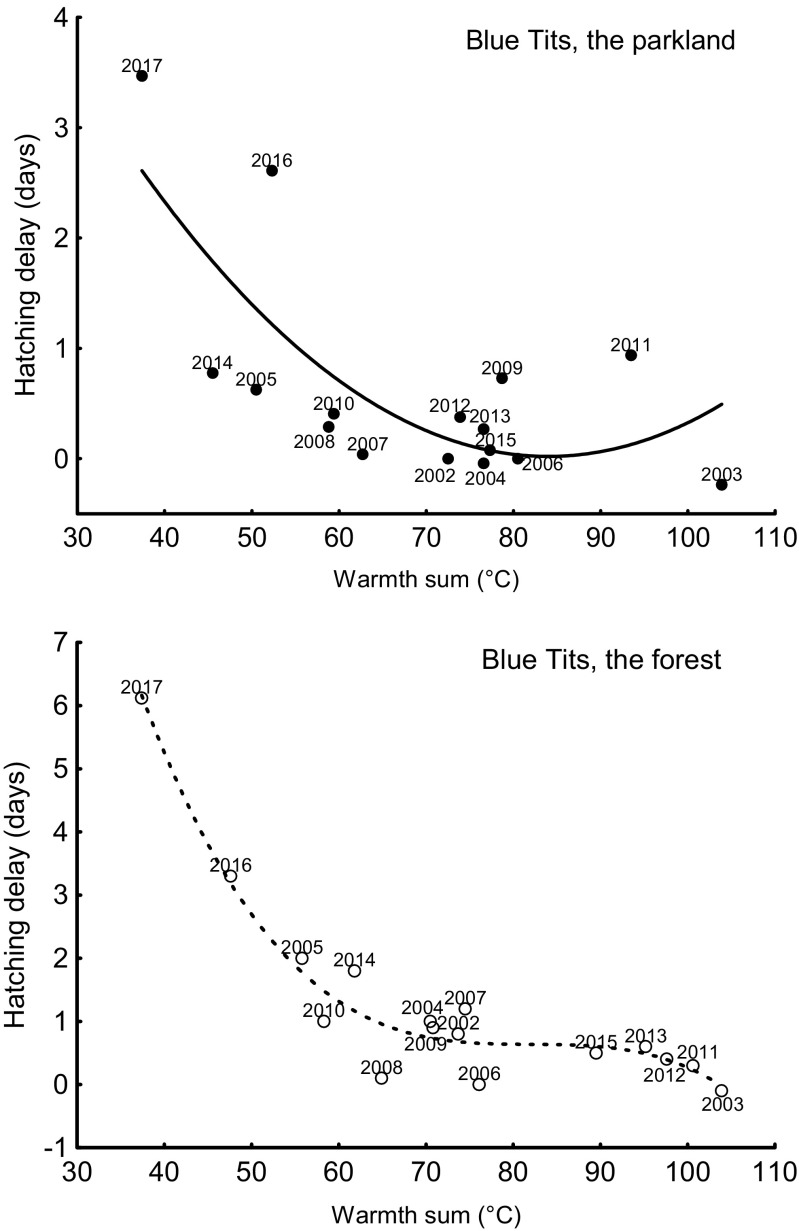
Relationship between hatching delay (days) and the sum of the mean daily temperatures for the 7 days starting on the 4th day since the first egg date (warmth sum, °C) for blue tits in the urban parkland area (quadratic, filled circles) and in the forest area (cubic, open circles) (seasons 2002–2017)

The main period of the temperature drop during the mid-laying-early-incubating time of tits in Łódź in April 2017 lasted for about 5 days and was characterized by the mean ambient temperature ca. 2–3 °C, with no snow cover (Fig. 1). We define a cold spell as a sudden drop in ambient temperature for a relatively short period of time and extreme weather events as weather conditions that cause the biological response to be in the 5% of most extreme values of the biological response variable (Altwegg et al. 2017; van de Pol et al. 2017). The biological response variable was hatching delay, which occurred in most extreme form in 2017 in both study areas and for both tit species (Figs. 2, 3, and 4). The local temperatures (average annual temperature) for Łódź were obtained from TuTiempo.net climate database for Łódź (http://www.tutiempo.net/en/Climate/LODZ/124650.htm and https://en.tutiempo.net/climate/ws-121055.html). Following García-Navas and Sanz (2011), we calculated the expected hatching date as follows: first egg date + clutch size + 12 (incubation in these species normally lasts 13 days and female usually starts to incubate 1 day before completing the clutch (García-Navas and Sanz 2011). The difference between this date and observed hatching date was taken as hatching delay (negative values equals hatching occurred before expected and positive values equals a delay). In all calculations, the mean laying dates were expressed as days from 1 March. Following Perrins and McCleery (1989) and Glądalski et al. (2014), we calculated mid-laying-early-incubating warmth sums (mid-laying temperatures are crucial for hatching delays; García-Navas and Sanz 2011; Cresswell and McCleery 2003), as the sum of the mean daily temperatures for the 7 days starting on the 4th day since the first egg date (first egg date + 4), to characterize thermal conditions during egg laying.

We computed general linear models to examine effects of year and site factors (factorial design ANOVA) on hatching delays, assuming a Gaussian error structure. Separate models for blue tits and great tits were fit. Because interactions of the main factors were significant, the full models were presented. To check if the relationship between hatching delays and thermal conditions (mid-laying-early-incubating warmth sums) was linear or non-linear, we calculated polynomial regressions with cubic and quadratic terms. We used *t* tests for the cubic and quadratic terms to delete non-significant terms. We also present adjusted values of *r*^2^ to evaluate the fit of the regressions. STATISTICA 12 (StatSoft Inc 2014) was applied to perform all computing and to produce charts.

## Results

Mid-laying-early-incubating warmth sums were extremely low in 2017 in comparison with the values for the preceding 15 years and, what is crucial, this large temperature drop happened at the time of egg laying (Fig. 2). The yearly mean hatching delay was negatively correlated with the warmth sums over the study years at both study areas for both tit species: great tits in the urban parkland and in the forest, and blue tits in the urban parkland and in the forest (Table 1, Figs. 3 and 4). In all cases, the relationship was non-linear and it suggests that the hatching delays for low temperatures are disproportionately larger than for average conditions.

**Table 1:** Summary of negative, linear (*x*), and non-linear relationships (*x*^2^—quadratic term, *x*^3^—cubic term) between hatching delay (days) and the sum of the mean daily temperatures (*x* expressed in °C) for the 7 days starting on the 4th day since the first egg date for great tits and blue tits in the parkland area and in the forest area (seasons 2002–2017)

	*b*	SE *b*	*t* (12)	*p*	*r*^2^ (adjusted)	*F* (3, 12)	*p*
Great tits, the forest							
Intercept	13.80	2.06	6.69	< 0.001	0.89	39.84	< 0.001
*x*	− 0.46	0.10	− 4.67	< 0.001			
*x*^2^	0.01	0.001	3.63	0.004			
*x*^3^	< 0.001	< 0.001	− 3.04	0.010			
Great tits, the parkland							
Intercept	26.88	4.97	5.41	< 0.001	0.88	36.20	< 0.001
*x*	− 0.97	0.24	− 4.11	0.002			
*x*^2^	0.01	0.004	3.23	0.007			
*x*^3^	< 0.001	< 0.001	− 2.64	0.022			
Blue tits, the forest							
Intercept	34.74	5.61	6.20	< 0.001	0.92	59.62	< 0.001
*x*	− 1.25	0.26	− 4.76	< 0.001			
*x*^2^	0.02	0.004	3.90	0.002			
*x*^3^	< 0.001	< 0.001	− 3.36	0.006			
Blue tits, the parkland							
Intercept	23.67	7.99	2.96	0.012	0.59	8.08	0.003
*x*	− 0.91	0.37	− 2.50	0.028			
*x*^2^	0.01	0.01	2.20	0.049			
*x*^3^	< 0.001	< 0.001	− 1.98	ns			

**Fig. 5:**
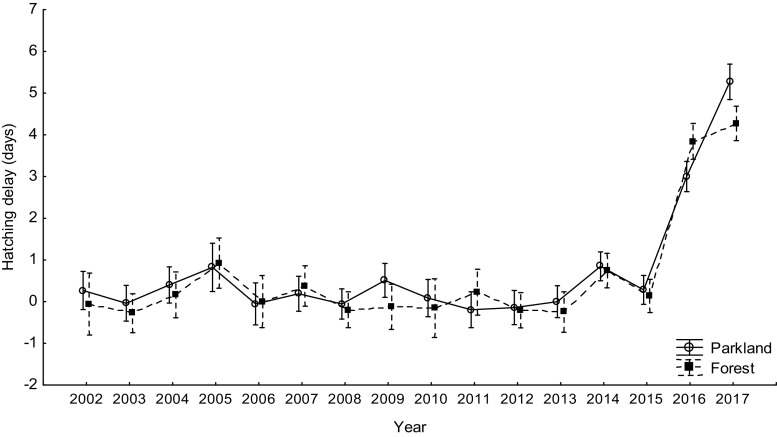
Mean hatching delay in the great tit in the urban parkland and in the forest study areas (2002–2017). Mean hatching delay is presented as average ± 95% confidence intervals

**Fig. 6:**
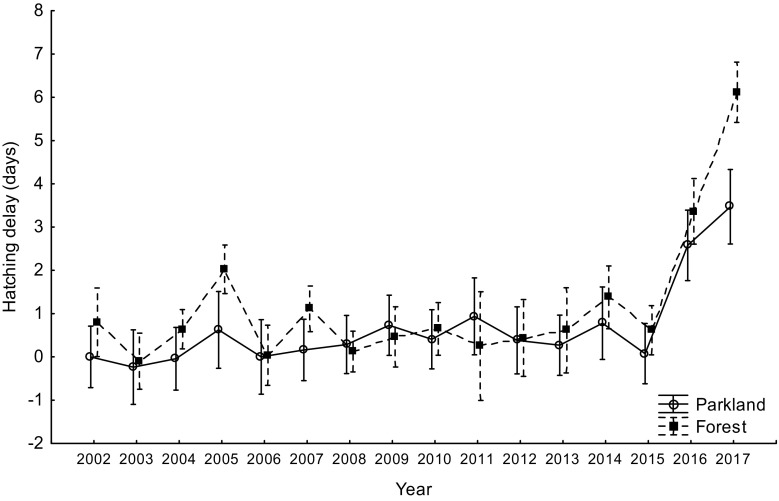
Mean hatching delay in the blue tit in the urban parkland and in the forest study areas (2002–2017). Mean hatching delay is presented as average ± 95% confidence intervals

In both tit species, the largest hatching delays occurred in 2017: mean for great tits 5.27 ± 4.4 SD days in the urban parkland and 4.27 ± 2.24 SD days in the forest and for blue tits 3.47 ± 2.10 SD days in the urban parkland and 6.12 ± 5.91 SD in the forest (Figs. 5 and 6). In the great tit, hatching delay was affected by a significant interaction between study area and year (Table 2). The interaction results from the fact that in most years, there is no interhabitat difference in the hatching delay, whereas there is a significant difference in the extreme year 2017 (Fig. 5). Although the hatching delay is large in both 2016 and 2017, only in 2017 is it so clear (Fig. 5). The difference is likely to result from a small difference in the breeding phenology of tits between the park area and the forest area. In the blue tit, hatching delay was also affected by a significant interaction between study area and year (Table 2). The interaction in blue tits results from the difference in hatching delay between habitats being exceptionally large in 2017, but in reverse direction in comparison to great tits (Figs. 5 and 6). This reverse direction may result from a between-species difference in phenology. In both great tits and blue tits, hatching delays that occurred in 2017 were exceptionally long, with the delays in both tit species in 2016 and in blue tits in 2005 also being substantially long (Table 3).

**Table 2:** Summary of general linear models of hatching delay in great tits and blue tits in relation to the effects of year and habitat (2002–2017)

Response variable	Factor	*F*	*df*	*p*
Hatching delay	Year	86.06	15	< 0.001
(great tits)	Habitat	1.65	1	0.20
	Year × habitat	1.76	15	0.036
Hatching delay (blue tits)	Year	20.62	15	< 0.001
	Habitat	13.78	1	< 0.001
	Year × habitat	1.88	15	0.022

**Table 3:** Summary of Tukey’s post hoc effects of year and habitat interaction analysis on hatching delay (2002–2017) in great tits (left-down) and in blue tits (right-up) in the urban parkland and in the forest (−*p* > 0.05; *p < 0.05; **p < 0.01; ***p < 0.001)

Year	Area	02/P	02/F	03/P	03/F	04/P	04/F	05/P	05/F	06/P	06/F	07/P	07/F	08/P	08/F	09/P	09/F	10/P	10/F	11/P	11/F	12/P	12/F	13/P	13/F	14/P	14/F	15/P	15/F	16/P	16/F	17/P	17/F
02	P		–	–	–	–	–	–	**	–	–	–	–	–	–	–	–	–	–	–	–	–	–	–	–	–	–	–	–	***	***	***	***
02	F	–		–	–	–	–	–	–	–	–	–	–	–	–	–	–	–	–	–	–	–	–	–	–	–	–	–	–	–	***	***	***
03	P	–	–		–	–	–	–	**	–	–	–	–	–	–	–	–	–	–	–	–	–	–	–	–	–	–	–	–	***	***	***	***
03	F	–	–	–		–	–	–	***	–	–	–	–	–	–	–	–	–	–	–	–	–	–	–	–	–	–	–	–	***	***	***	***
04	P	–	–	–	–		–	–	**	–	–	–	–	–	–	–	–	–	–	–	–	–	–	–	–	–	–	–	–	***	***	***	***
04	F	–	–	–	–	–		–	–	–	–	–	–	–	–	–	–	–	–	–	–	–	–	–	–	–	–	–	–	*	***	***	***
05	P	–	–	–	–	–	–		–	–	–	–	–	–	–	–	–	–	–	–	–	–	–	–	–	–	–	–	–	–	**	**	***
05	F	–	–	–	–	–	–	–		*	**	–	*	*	***	–	–	–	–	–	–	–	–	*	–	–	–	**	–	–	–	–	***
06	P	–	–	–	–	–	–	–	–		–		–	–	–	–	–	–	–	–	–	–	–	–	–	–	–	–	–	**	***	***	***
06	F	–	–	–	–	–	–	–	–	–		–	–	–	–	–	–	–	–	–	–	–	–	–	–	–	–	–	–	**	***	***	***
07	P	–	–	–	–	–	–	–	–	–	–		–	–	–	–	–	–	–	–	–	–	–	–	–	–	–	–	–	**	***	***	***
07	F	–	–	–	–	–	–	–	–	–	–	–		–	–	–	–	–	–	–	–	–	–	–	–	–	–	–	–	–	***	***	***
08	P	–	–	–	–	–	–	–	–	–	–	–	–		–	–	–	–	–	–	–	–	–	–	–	–	–	–	–	**	***	***	***
08	F	–	–	–	–	–	–	–	–	–	–	–	–	–		–	–	–	–	–	–	–	–	–	–	–	–	–	–	***	***	***	***
09	P	–	–	–	–	–	–	–	–	–	–	–	–	–	–		–	–	–	–	–	–	–	–	–	–	–	–	–	–	***	***	***
09	F	–	–	–	–	–	–	–	–	–	–	–	–	–	–	–		–	–	–	–	–	–	–	–	–	–	–	–	*	***	***	***
10	P	–	–	–	–	–	–	–	–	–	–	–	–	–	–	–	–		–	–	–	–	–	–	–	–	–	–	–	*	***	***	***
10	F	–	–	–	–	–	–	–	–	–	–	–	–	–	–	–	–	–		–	–	–	–	–	–	–	–	–	–	–	***	***	***
11	P	–	–	–	–	–	–	–	–	–	–	–	–	–	–	–	–	–	–		–	–	–	–	–	–	–	–	–	–	*	*	***
11	F	–	–	–	–	–	–	–	–	–	–	–	–	–	–	–	–	–	–	–		–	–	–	–	–	–	–	–	–	**	**	***
12	P	–	–	–	–	–	–	–	–	–	–	–	–	–	–	–	–	–	–	–	–		–	–	–	–	–	–	–	*	***	***	***
12	F	–	–	–	–	–	–	–	–	–	–	–	–	–	–	–	–	–	–	–	–	–		–	–	*	–	–	–	–	***	***	***
13	P	–	–	–	–	–	–	–	–	–	–	–	–	–	–	–	–	–	–	–	–	–	–		–	–	–	–	–	**	***	***	***
13	F	–	–	–	–	–	–	–	–	–	–	–	–	–	–	–	–	–	–	–	–	–	–	–		–	–	–	–	–	**	**	***
14	P	–	–	–	*	–	–	–	–	–	–	–	–	–	–	–	–	–	–	–	–	–	*	–	–		–	–	–	–	**	**	***
14	F	–	–	–	–	–	–	–	–	–	–	–	–	–	–	–	–	–	–	–	–	–	–	–	–	–		–	–	–	–	–	***
15	P	–	–	–	–	–	–	–	–	–	–	–	–	–	–	–	–	–	–	–	–	–	–	–	–	–	–		–	**	***	***	***
15	F	–	–	–	–	–	–	–	–	–	–	–	–	–	–	–	–	–	–	–	–	–	–	–	–	–	–	–		*	***	***	***
16	P	***	***	***	***	***	***	***	***	***	***	***	***	***	***	***	***	***	***	***	***	***	***	***	***	***	***	***	***		–	–	***
16	F	***	***	***	***	***	***	***	***	***	***	***	***	***	***	***	***	***	***	***	***	***	***	***	***	***	***	***	***	–		–	***
17	P	***	***	***	***	***	***	***	***	***	***	***	***	***	***	***	***	***	***	***	***	***	***	***	***	***	***	***	***	***	***		***
17	F	***	***	***	***	***	***	***	***	***	***	***	***	***	***	***	***	***	***	***	***	***	***	***	***	***	***	***	***	***	–	–	

## Discussion

Currently, many avian species in Europe migrate and breed earlier as a result of higher temperatures caused by global climate changes (DeLeon et al. 2011; Fletcher et al. 2013; Charmantier and Gienapp 2014). The great tit and the blue tit are among such earlier breeding species (Bauer et al. 2010). Our previous studies show that extreme phenomena may act in opposition to general trends (Glądalski et al. 2014, 2016a)—the generally warmer and earlier springs do not exclude spells of exceptionally unfavorable weather occasionally. In the present study, hatching delays of great tits and blue tits were highly correlated with temperatures during the mid-laying-early-incubating period. This study shows that the large temperature drop during the laying period in 2017 caused extreme hatching delays in both tit species at both our study areas. In all cases, the relationship was non-linear; therefore, it suggests that the hatching delays for low temperatures are disproportionately larger than for average conditions. It is difficult to tell whether hatching delay immense flexibility is a unique feature of great tits and blue tits. Several studies analyzed the potential of tits for adjusting the interval between laying and hatching date and excluding Cresswell and McCleery (2003), all those studies analyze only 1–3 breeding seasons (Monrós et al. 1998; Naef-Daenzer et al. 2004; García-Navas and Sanz 2011; Kluen et al. 2011). Cresswell and McCleery (2003) suggested that birds increase their fitness by synchronizing their production of offspring with a peak of food abundance (in the case of tits, caterpillars are the optimal food for nestlings). This synchronization may be accomplished by varying the moment of clutch initiation (and this is very flexible in tits), but temperature characteristics during the egg production phase may delay or accelerate the caterpillar peak. Another way of synchronization may be downsizing of the clutch, laying gaps or delaying/accelerating the onset of incubation (Tomás 2015). Tomás (2015) even suggests that the hatching date should be analyzed as a more proper phenological trait than the laying date because of those mechanisms that allow a female to synchronize her production of offspring with a peak of food abundance. Laying gaps may be caused also by food shortage (a low temperature inhibits activity of insects (Mellanby 1939; Bale 2002) and may reduce prey accessibility for birds) or increased costs of thermoregulation during egg laying period (Nilsson and Svensson 1993a, b). But the “strategy” and the “constraint” hypotheses are not mutually exclusive, and probably both energetic limitations and behavioral decisions contributed to the observed hatching delays (Naef-Daenzer et al. 2004). Monrós et al. (1998) conclude that some delays in the hatching date could be beneficial for parents and offspring, since they seemed to allow for a better adjustment to changes in environmental conditions.

The difference in hatching delay between the study areas and years (thus also interaction) in both tit species may be caused by a difference in phenology of great tits and blue tits in combination with a difference in phenology of the study habitats (blue tits tend to initiate clutch mean 1.5 days earlier than great tits in the parkland area and mean 2.5 earlier in the forest area, unpublished data). Urban environments are usually associated with earlier clutches in tits (Bańbura and Bańbura 2012; Seress and Liker 2015; Marini et al. 2017). Taxonomic composition of tree flora in the parkland results in earlier leafing—buds and thus larvae on poplars and birches (the parkland) appear earlier than on oaks (the forest) (Glądalski et al. 2015; Wawrzyniak et al. 2015). The leafing phenology directly influences the occurrence of caterpillars (the most important component of the diet of chicks, sometimes supplemented by spiders and other insects (Blondel et al. 1993)). Those shifts in timing may affect the hatching delay because when the drop of a temperature happens birds in one study area may be a few days later in clutch completion or vice versa.

This paper is based on the occurrence of a cold spell as a natural experiment in which we could not control environmental conditions and properly ascribe initiated clutches to treatments. This results from the fact that it is obviously not possible to experimentally manipulate the ambient temperature in the field. We see two ways in which experiments capable of identifying more precisely at least some proximate mechanisms underlying hatching delays could be performed. One way would be to use indoor aviaries to manipulate thermal conditions during the egg laying stage of breeding. The other way, available in the field in the case of hole-nesting birds, would be experimental cooling of nestboxes at the time of egg laying. As far as we know, such experimental cooling has only been used to study different effects of thermal conditions for incubation so far (e.g., Alvarez and Barba 2014).

## References

[CR1] Altwegg R, Visser V, Bailey LD, Erni B (2017). Learning from single extreme events. Philos Trans R Soc Lond B.

[CR2] Alvarez E, Barba E (2014). Behavioural responses of great tits to experimental manipulation of nest temperature during incubation. Ornis Fenn.

[CR3] Bailey LD, van de Pol M (2016). Tackling extremes: challenges for ecological and evolutionary research on extreme climatic events. J Anim Ecol.

[CR4] Bale JS (2002). Insects and low temperatures: from molecular biology to distributions and abundance. Philos Trans R Soc Lond B.

[CR5] Bańbura J, Bańbura M (2012). Blue tits Cyanistes caeruleus and great tits Parus major as urban habitat breeders. Int Stud Sparrows.

[CR6] Bateman BL, Pidgeon AM, Radeloff VC, Allstadt AJ, Akçakaya HR, Thogmartin WE, Vavrus SJ, Heglund PJ (2015). The importance of range edges for an irruptive species during extreme weather events. Landsc Ecol.

[CR7] Bauer Z, Trnka M, Bauerová J, Mozný M, Stepánek P, Bartosová L, Zalud Z (2010). Changing climate and the phenological response of great tit and collared flycatcher populations in floodplain forest ecosystems in Central Europe. Int J Biometeorol.

[CR8] Bleu J, Agostini S, Biard C (2017). Nest-box temperature affects clutch size, incubation initiation, and nestling health in great tits. Behav Ecol.

[CR9] Blondel J, Dias PC, Maistre M, Perret P (1993). Habitat heterogeneity and life-history variation of Mediterranean blue tits (Parus caeruleus). Auk.

[CR10] Both C, Visser ME (2005). The effect of climate change on the correlation between avian life-history traits. Glob Chang Biol.

[CR11] Buckley LB, Huey RB (2016). Temperature extremes: geographic patterns, recent changes, and implications for organismal vulnerabilities. Glob Chang Biol.

[CR12] Charmantier A, Gienapp P (2014). Climate change and timing of avian breeding and migration: evolutionary versus plastic changes. Evol Appl.

[CR13] Charmantier A, McCleery RH, Cole LR, Perrins C, Kruuk LE, Sheldon BC (2008). Adaptive phenotypic plasticity in response to climate change in a wild bird population. Science.

[CR14] Cresswell W, McCleery RH (2003). How great tits maintain synchronization of their hatch date with food supply in response to long-term variability in temperature. J Anim Ecol.

[CR15] Cucco M, Grenna M, Pellegrino (2017). Egg characteristics in relation to skipped days of laying in the grey partridge. Avian Biol Res.

[CR16] DeLeon RL, DeLeon EE, Rising GR (2011). Influence of climate change on avian migrants’ first arrival dates. Condor.

[CR17] Donnelly A, Yu R (2017). The rise of phenology with climate change: an evaluation of IJB publications. Int J Biometeorol.

[CR18] Fletcher K, Howarth D, Kirby A, Dunn R, Smith A (2013). Effect of climate change on breeding phenology, clutch size and chick survival of an upland bird. Ibis.

[CR19] García-Navas V, Sanz JJ (2011). Short-term alterations in songbird breeding schedule lead to better synchronization with food availability. Auk.

[CR20] Gaughan JB, Lees AM, Sejian V (2017) Sixty years of animal biometeorology. Int J Biometeorol. 10.1007/s00484-017-1459-1 10.1007/s00484-017-1459-1 29058080

[CR21] Glądalski M, Bańbura M, Kaliński A, Markowski M, Skwarska J, Wawrzyniak J, Zieliński P, Bańbura J (2014). Extreme weather event in spring 2013 delayed breeding time of great tit and blue tit. Int J Biometeorol.

[CR22] Glądalski M, Bańbura M, Kaliński A, Markowski M, Skwarska J, Wawrzyniak J, Zieliński P, Cyżewska I, Bańbura J (2015). Inter-annual and inter-habitat variation in breeding performance of blue tits (Cyanistes caeruleus) in central Poland. Ornis Fenn.

[CR23] Glądalski M, Bańbura M, Kaliński A, Markowski M, Skwarska J, Wawrzyniak J, Zieliński P, Bańbura J (2016). Effects of extreme thermal conditions on plasticity in breeding phenology and double-bloodedness of great tits and blue tits in central Poland in 2013 and 2014. Int J Biometeorol.

[CR24] Glądalski M, Bańbura M, Kaliński A, Markowski M, Skwarska J, Wawrzyniak J, Zieliński P, Cyżewska I, Mańkowska D, Bańbura J (2016). Effects of human-related disturbance on breeding success of urban and non-urban blue tits (Cyanistes caeruleus). Urban Ecosyst.

[CR25] Glądalski M, Bańbura M, Kaliński A, Markowski M, Skwarska J, Wawrzyniak J, Zieliński P, Cyżewska I, Bańbura J (2017). Differences in the breeding success of blue tits (Cyanistes caeruleus) between a forest and an urban area: a long-term study. Acta Ornithol.

[CR26] Goodenough AE, Hart AG, Elliot SL (2011). What prevents phenological adjustment to climate change in migrant bird species? Evidence against the Barrival constraint hypothesis. Int J Biometeorol.

[CR27] Indykiewicz P (2015). Egg losses caused by cold snap in the black-headed gull, Chroicocephalus ridibundus L. Pol J Ecol.

[CR28] Jenouvrier S (2013). Impacts of climate change on avian populations. Glob Chang Biol.

[CR29] Jentsch A, Kreyling J, Beierkuhnlein C (2007). A new generation of climate-change experiments: events, not trends. Front Ecol Environ.

[CR30] Hinks AE, Cole EF, Daniels KJ, Wilkin TA, Nakagawa S, Sheldon BC (2015). Scale-dependent phenological synchrony between songbirds and their caterpillar food source. Am Nat.

[CR31] Kluen E, de Heij ME, Brommer JE (2011). Adjusting the timing of hatching to changing environmental conditions has fitness costs in blue tits. Behav Ecol Sociobiol.

[CR32] Lambrechts M, Adriaensen F, Ardia DR, Artemyev AV, Atiénzar F, Bańbura J, Barba E, Bouvier J-C, Camprodon J (2010). The design of artificial nestboxes for the study of secondary holenesting birds: a review of methodological inconsistencies and potential biases. Acta Ornithol.

[CR33] Lee JK, Lima SL (2017). Egg viability as a determinant of clutch size in birds: a basic analysis. Avian Biol Res.

[CR34] Mainwaring MC, Barber I, Deeming DC, Pike DA, Roznik EA, Hartley IR (2017). Climate change and nesting behaviour in vertebrates: a review of the ecological effects and potential for adaptive responses. Biol Rev.

[CR35] Mainwaring MC, Hartley IR (2016). Local weather conditions have complex effects on the growth of blue tit nestlings. J Therm Biol.

[CR36] Marini KLD, Otter KA, LaZerte SE, Reudink MW (2017). Urban environments are associated with earlier clutches and faster nestling feather growth compared to natural habitats. Urban Ecosyst.

[CR37] Marciniak B, Nadolski J, Nowakowska M, Loga B, Bańbura J (2007). Habitat and annual variation in arthropod abundance affects blue tit Cyanistes caeruleus reproduction. Acta Ornithol.

[CR38] Marrot P, Garant D, Charmantier A (2017). Multiple extreme climatic events strengthen selection for earlier breeding in a wild passerine. Phil Trans R Soc Lond B.

[CR39] Martinuzzi S, Allstadt AJ, Bateman BL, Heglund PJ, Pidgeon AM, Thogmartin Vavrus SJ, Radeloff VC (2016). Future frequencies of extreme weather events in the National Wildlife Refuges of the conterminous U.S. Biol Conserv.

[CR40] Massa B, Cusimano CA, Margagliotta B, Galici R (2011). Reproductive characteristics and differential response to seasonal temperatures of blue and great tits (*Cyanistes caeruleus* & *Parus major*) in three neighbouring Mediterranean habitats. Rev Écol (Terre Vie).

[CR41] Mellanby K (1939). Low temperature and insect activity. Proc R Soc Lond B.

[CR42] Monrós IS, Belda EJ, Barba E (1998). Delays of the hatching dates in great tits Parus major: effects on breeding performance. Ardea.

[CR43] Naef-Daenzer L, Nager RG, Keller LF, Naef-Daenzer B (2004). Are hatching delays a cost or a benefit for great tit Parus major parents?. Ardea.

[CR44] Nilsson J-A, Svensson E (1993). Energy constraints and ultimate decisions during egg-laying in the blue tit. Ecology.

[CR45] Nilsson J-A, Svensson E (1993). The frequency and timing of laying gaps. Ornis Scand.

[CR46] Otto FEL (2015). Climate change: attribution of extreme weather. Nat Geosci.

[CR47] Perrins CM, McCleery RH (1989). Laying dates and clutch size in the great tit. Wilson Bull.

[CR48] Perrins CM (1991). Tits and their caterpillar food supply. Ibis.

[CR49] Perrins CM (1996). Eggs, egg formation and the timing of breeding. Ibis.

[CR50] Pipoly I, Bókony V, Seress G, Szabó K, Liker A (2013). Effects of extreme weather on reproductive success in a temperate-breeding songbird. PLoS One.

[CR51] Rodríguez S, Diez-Méndez D, Barba E (2016). Negative effects of high temperatures during development on immediate post-fledging survival in great tits Parus major. Acta Ornithol.

[CR52] Seress G, Liker A (2015). Habitat urbanization and its effects on birds. Acta Zool Acad Sci Hung.

[CR53] Sheridan S, Allen MJ (2017). Sixty years of the International Journal of Biometeorology. Int J Biometeorol.

[CR54] StatSoft Inc (2014) STATISTICA (data analysis software system), version 12. URL: http://www.statsoft.com

[CR55] Stearns SC (1992). The evolution of life histories.

[CR56] Tobolka M, Zolnierowicz KM, Reeve NF (2015). The effect of extreme weather events on breeding parameters of the white stork Ciconia ciconia. Bird Study.

[CR57] Tomás G (2015). Hatching date vs laying date: what should we look at to study avian optimal timing of reproduction?. J Avian Biol.

[CR58] Ummenhofer CC, Meehl GA (2017). Extreme weather and climate events with ecological relevance: a review. Philos Trans R Soc Lond B.

[CR59] van de Pol M, Jenouvrier S, Cornelinnen JHC, Visser ME (2017). Behavioural, ecological and evolutionary responses to extreme climatic events: challenges and directions. Philos Trans R Soc B.

[CR60] van Noordwijk AJ, McCleery RH, Perrins CM (1995). Selection for the timing of great tit breeding in relation to caterpillar growth and temperature. J Anim Ecol.

[CR61] Vaugoyeau M, Meylan S, Biard C (2017). How does an increase in minimum daily temperatures during incubation influence reproduction in the great tit Parus major?. J Avian Biol.

[CR62] Wawrzyniak J, Kaliński A, Glądalski M, Bańbura M, Markowski M, Skwarska J, Zieliński P, Cyżewska I, Bańbura J (2015). Long-term variation in laying date and clutch size of the great tit Parus major in central Poland: a comparison between urban parkland and deciduous forest. Ardeola.

[CR63] Wesołowski T, Cholewa M, Hebda G, Maziarz M, Rowiński P (2016). Immense plasticity of timing of breeding in a sedentary forest passerine, Poecile palustris. J Avian Biol.

[CR64] Whitehouse MJ, Harrison NM, Mackenzie J, Hinsley SA (2013). Preferred habitat of breeding birds may be compromised by climate change: unexpected effects of an exceptionally cold, wet spring. PLoS One.

